# In Silico and In Vivo Investigation of the Anti-Hyperglycemic Effects of Caffeic Acid

**DOI:** 10.1021/acsomega.4c11062

**Published:** 2025-04-01

**Authors:** Ratnawati Ratnawati, Muhammad Aswad, Jumriani Jumriani, Anggun Nurhidayah, Muhammad Rayza Azmin, Filmaharani Filmaharani, Alfreds Roosevelt, Widya Hardiyanti, Nadila Pratiwi Latada, Mukarram Mudjahid, Firzan Nainu

**Affiliations:** † Postgraduate Program in Pharmacy, Faculty of Pharmacy, Hasanuddin University, Tamalanrea, Makassar 90245, Indonesia; ‡ Department of Pharmaceutical Science and Technology, Faculty of Pharmacy, Hasanuddin University, Tamalanrea, Makassar 90245, Indonesia; § Unhas Fly Research Group, Faculty of Pharmacy, Hasanuddin University, Tamalanrea, Makassar 90245, Indonesia; ∥ Department of Pharmacy, Faculty of Pharmacy, Hasanuddin University, Tamalanrea, Makassar 90245, Indonesia

## Abstract

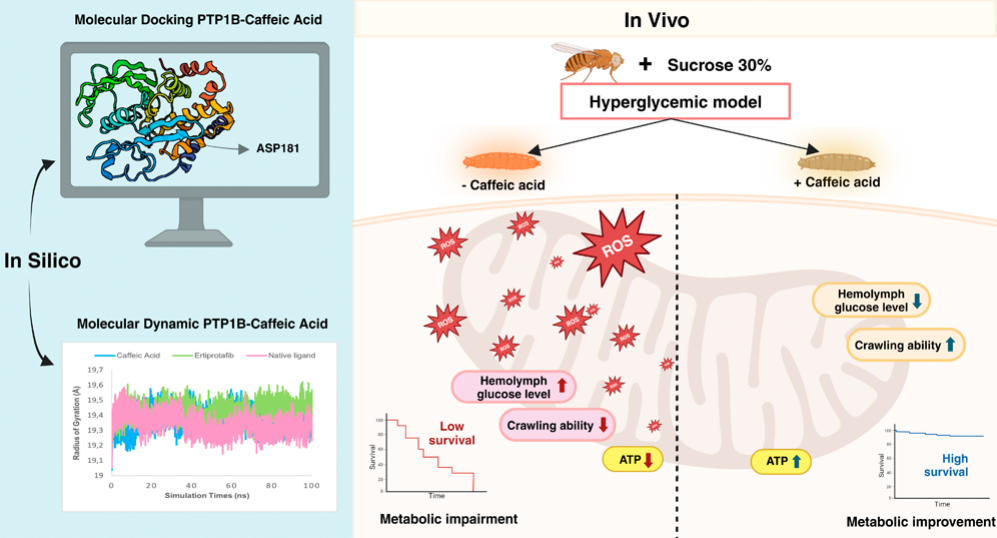

Hyperglycemia, characterized by elevated blood glucose levels, is a major risk factor for diabetes mellitus and its complications. While conventional therapies are effective, they are often associated with side effects and high costs, necessitating alternative strategies. This study evaluates the potential of caffeic acid (CA), a phenolic compound with reported antihyperglycemic properties, using both in silico and in vivo approaches. Molecular docking simulations revealed that CA demonstrates a strong binding affinity to protein tyrosine phosphatase 1B (PTP1B), a critical enzyme in glucose metabolism, with superior interaction profiles compared to the reference drug, ertiprotafib. In the in vivo studies, a *Drosophila melanogaster* model was used to investigate the effects of CA under hyperglycemic conditions induced by a high-sugar diet. Treatment with CA, particularly at a concentration of 500 μM, significantly reduced hemolymph glucose levels and improved several physiological and behavioral parameters, including survival rates, body size, body weight, and larval movement. Furthermore, gene expression analysis demonstrated that CA modulates key metabolic and stress-related pathways, enhancing glucose homeostasis and reducing metabolic stress. These findings highlight the dual utility of in silico and in vivo methodologies in elucidating the antihyperglycemic potential of CA. The results support the development of CA as a cost-effective and ethically viable therapeutic candidate with implications for diabetes management in resource-limited settings.

## 1 Introduction

Hyperglycemia, defined as abnormally elevated blood glucose levels, is a hallmark of metabolic dysregulation commonly associated with diabetes mellitus. Data from the International Diabetes Federation (IDF) in 2021 highlighted the growing prevalence of hyperglycemia among adults, with an estimated 537 million individuals aged 20–79 years living with diabetes. This figure is projected to rise to 643 million by 2030^1^ and approximately 1.31 billion by 2050.^2^ This alarming trend emphasizes the critical need for improved strategies to manage and mitigate complications arising from chronic hyperglycemia.

Hyperglycemia plays a pivotal role in the development of diabetes-related complications, including cardiovascular disease, neuropathy, nephropathy, retinopathy, and diabetic ketoacidosis, all of which can lead to increased mortality rates.^3,4^ Consequently, maintaining optimal blood glucose levels is essential to reducing the risk of these adverse outcomes. In the management of type 2 diabetes mellitus (T2DM), several molecular targets have been identified as key therapeutic focuses, including α-glucosidase, dipeptidyl peptidase-4 (DPP-4), peroxisome proliferator-activated receptor gamma (PPARγ), and protein tyrosine phosphatase 1B (PTP1B).^5,6^ These targets represent promising avenues for the development of more effective treatments aimed at addressing the global burden of diabetes and its complications.

Currently, a variety of conventional drugs are available for the treatment of diabetes mellitus. Among them, α-glucosidase inhibitors, such as acarbose and miglitol, function by slowing or reducing glucose absorption in the digestive tract, effectively lowering postprandial blood glucose levels and mitigating hyperglycemia.^5,7^ Similarly, DPP-4 inhibitors play a critical role in maintaining glucose homeostasis by enhancing insulin secretion and suppressing glucagon release.^8^ PPARγ agonists represent another therapeutic class, improving insulin sensitivity and facilitating a glucose uptake in the skeletal muscle and adipose tissues.^9^ Additionally, the inhibition of PTP1B activity has been demonstrated to enhance insulin sensitivity and promote glucose uptake, particularly in liver and muscle tissues, positioning it as a promising strategy for T2DM management.^10^ However, the long-term use of these medications is often associated with adverse side effects, including urinary tract infections, lactic acidosis, heart failure, and osteoporosis, in addition to significant financial costs.^11^ Consequently, there is a pressing need to explore and evaluate the antihyperglycemic potential of new compounds to develop more effective and cost-efficient therapeutic options.

In drug discovery and development research, tracing the activity of drug candidates through in silico, in vitro, and in vivo methods is a critical step to ensure their potential efficacy and safety before advancing to clinical trials.^12−15^ Computational advancements have accelerated drug discovery, with drug repositioning emerging as a cost- and time-efficient strategy by repurposing known compounds with established pharmacokinetic and toxicity profiles.^16^*In silico* techniques such as molecular docking and molecular dynamics (MD) simulations have emerged as indispensable tools in drug discovery. These methods enable the prediction of molecular interactions and the pharmacological activities of drug candidates against specific biological targets.^17,18^ Molecular docking identifies optimal ligand–protein binding orientations, while MD simulations provide insights into the structural dynamics and energetics of these interactions.^19^ These techniques, known for their time and cost efficiency, are particularly valuable in the early stages of drug development. In vitro assays are conducted to directly evaluate the biological activity and toxicity of drug candidates on cells or tissues outside a living organism, offering more concrete evidence regarding the drug’s effectiveness and safety profile.^14^ In contrast, in vivo studies, which involve testing within living organisms, are designed to assess the safety, efficacy, and pharmacokinetic and pharmacodynamic properties of drug candidates within complex biological systems.^15^ The integration of these three methodologies enables a systematic, efficient, and accurate approach to drug development, ensuring a comprehensive understanding of a compound’s potential before clinical application.

One compound widely recognized for its antihyperglycemic effects is caffeic acid (CA).^20−22^ Found in various foods and beverages such as coffee, kiwi, carrots, tomatoes, and honey,^23,24^ CA offers multiple health benefits, including antihypertensive,^25^ anti-inflammatory,^26^ immunomodulatory effect,^27^ and lowers blood glucose levels.^28^ Numerous in silico studies have identified the activity of CA against molecular targets such as α-glucosidase and DPP-4,^29^ while in vitro studies have demonstrated its ability to inhibit enzymes involved in glucose metabolism.^30^ Additionally, in vivo experiments using model organisms, including mice, have reported beneficial effects on insulin levels. However, the precise mechanisms by which CA influences cellular stress and apoptosis remain to be elucidated.^28^ These findings highlight the potential of CA to be further explored and developed as an antihyperglycemic agent.

Mammalian models have been traditionally employed in diabetes mellitus research. However, the growing emphasis on the 3R principle (replacement, reduction, and refinement) necessitates consideration of mammalian welfare. Consequently, researchers are increasingly exploring alternative model organisms for drug testing, with *Drosophila melanogaster* emerging as a prominent option. The use of *D. melanogaster* has many advantages such as having a genetic similarity with humans of about 75%, lower maintenance costs, and does not require ethical clearance. In addition, *D. melanogaster* also lacks a pancreas but has insulin receptors and insulin-producing cells that function analogously to the human pancreas. *D. melanogaster* synthesizes Drosophila insulin-like protein (DILP) through IPCs, which works similarly to human insulin.^31,32^ More importantly, previous studies have demonstrated that *D. melanogaster* can develop hyperglycemia and exhibit phenotypes resembling diabetes mellitus.^33,34^

This study aims to evaluate the potential antihyperglycemic activity of CA, with a specific focus on its effects on protein tyrosine phosphatase 1B (PTP1B). In addition to employing in silico methods, the research incorporates an in vivo approach using a hyperglycemia model in *D. melanogaster* larvae. Female *D. melanogaster* produce a high number of offspring within a short time frame, enabling large-scale experimentation, while their relatively short lifespan of 2–3 months makes them ideal for rapid studies.^35^ This alternative model organism has been widely used in studies on infection, metabolism, and immunity, demonstrating its versatility in biomedical research.^36−38^ By leveraging *D. melanogaster* as an in vivo model in conjunction with in silico molecular docking, this study aims to unravel the therapeutic potential of CA for managing hyperglycemia. The use of *D. melanogaster* also highlights the feasibility of conducting advanced genetic and molecular biology research in resource-limited settings such as in developing countries like Indonesia. This integrative approach not only contributes to a deeper understanding of the pharmacological effects of CA but also underscores the advantages of employing cost-effective and ethically sustainable methodologies in the study of hyperglycemia and related metabolic disorders.

## 2 Materials and Methods

### 2.1 Materials

In this study, CA (CAS RN.: 331–39–5) from the Tokyo Chemical Industry Co., Ltd. (TCI) and sucrose SMART-LAB (CAS no.: 57-50-1) were obtained from PT. Smart-Lab, Tangerang, Indonesia. Sucrose served as the key component in the preparation of a high-sugar diet (HSD) for *Drosophila*.

### 2.2 Preparation of CA Solution

The CA powder (0.0090 g) was dissolved in 5 mL of 70% ethanol to prepare a stock solution. Subsequently, a concentration series was created at 31.25, 125, and 500 μM.

### 2.3 Drosophila Stocks

**Figure 1:**
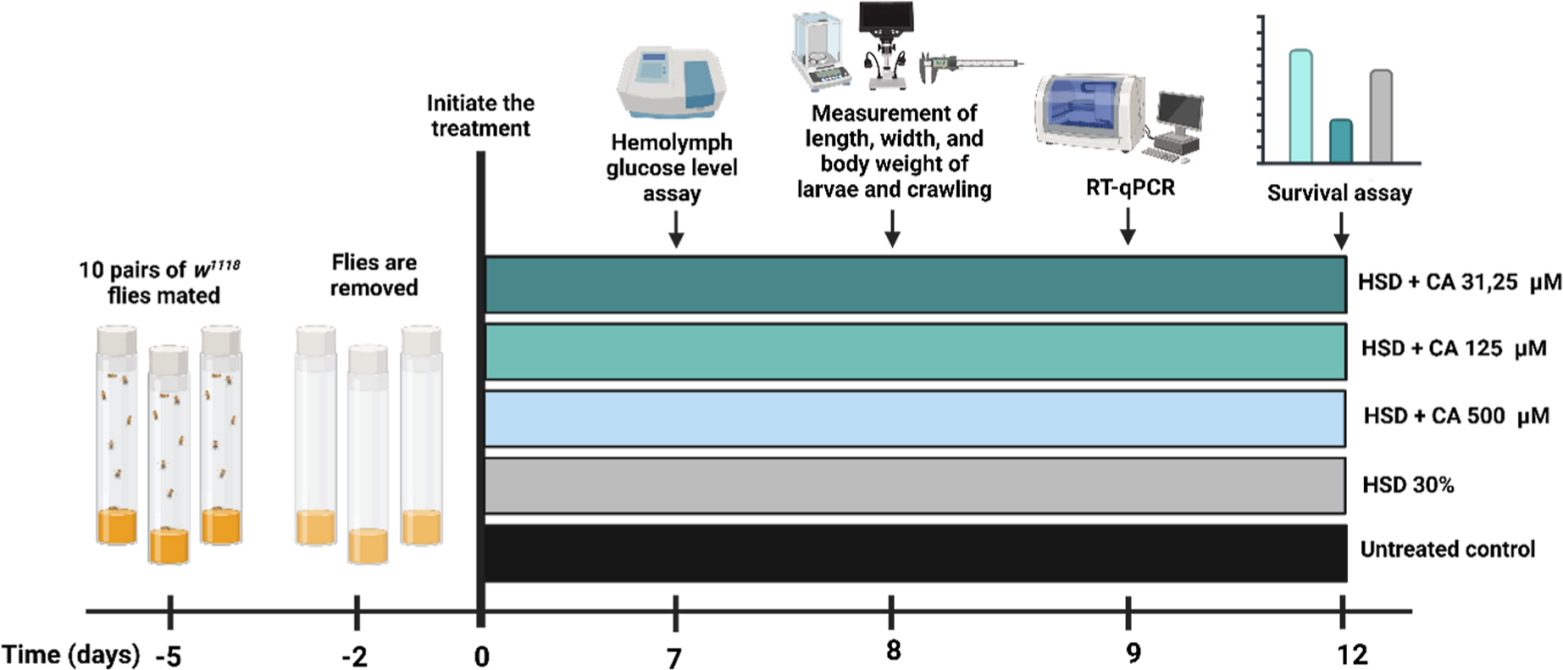
Experimental design used in this study. Five groups of 3rd instar larvae of *D. melanogaster* larvae were used to assess the effects of a HSD with or without CA treatment at varying concentrations (31.25, 125, and 500 μM). One group, reared without any treatment, served as the untreated control. Created with BioRender.com.

The *D. melanogaster* strain *w*^1118^ utilized as the model organism in this study was obtained from the Laboratory of Host Defense and Responses (Kanazawa University, Japan). Five pairs of *D. melanogaster* (5 males and 5 females) were maintained and bred in vials containing a standard feed at approximately 25 °C. The experimental design included five groups: a control group with no treatment, a HSD group, an HSD +31.25 μM CA group, an HSD +125 μM CA group, and an HSD +500 μM CA group. The composition of the fly food used in the experiment is detailed in Table 1. Each group included five replicates that were utilized for hemolymph glucose level testing, crawling assays, survival assessments, and gene expression analysis (Figure 1).

**Table 1:** Composition of the *D. melanogaster* Diet Used in This Study

compositions	normal diet (ND)	high sugar diet (HSD)
cornmeal	7.5 g	7.5 g
yeast	2.5 g	2.5 g
agar	0.9 g	0.9 g
sucrose	4.5 g	30 g^33,34^
propionic acid	400 μL	400 μL
methyl paraben	450 μL	450 μL
distilled water	ad 100 mL	ad 100 mL

### 2.4 Molecular Docking

#### 2.4.1 Ligand Preparation

The molecular structure of CA was obtained from the PubChem database. Using UCSF Chimera 1.17.3 (University of California, San Francisco, USA) software, the canonical SMILES format was converted into a protein data bank (PDB) file.^39^ To prepare the ligand for docking analysis, polar hydrogens were added, and Gasteiger charges were assigned using the “Minimize” tool in UCSF Chimera.^40^ This process minimized the ligand’s energy and optimized its geometry, ensuring suitability for subsequent docking studies.^41^

#### 2.4.2 Protein Preparation

The crystal structure of the target protein, protein tyrosine phosphatase 1B (PTP1B), was retrieved from the Research Collaboratory for Structural Bioinformatics PDB. Protein preparation was carried out using the “Dock Prep” tool in UCSF Chimera, which involved adding hydrogen atoms, repairing incomplete side chains, assigning charges, and removing solvent molecules. The processed protein structure was then saved in the PDB format to ensure compatibility with molecular docking software UCSF Chimera 1.17.3 integrated with AutoDock Vina and visualization tools Biovia Discovery Studio 2019 (Waltham, Massachusetts, USA).^40^

#### 2.4.3 Molecular Docking and Validation

The prepared ligand and protein structures were imported into UCSF Chimera, integrated with AutoDock Vina, for molecular docking analysis. A grid box was defined around the binding pocket of the protein, specifying its size and location to focus the docking simulations on the relevant region. The docking simulations were performed to predict the binding mode and affinity of CA for the protein target. Postdocking analyses were carried out to assess the interactions between the ligand and the protein, including hydrogen bonds, hydrophobic interactions, and binding affinities. The docking results were further validated and visualized using a Biovia Discovery Studio to confirm the accuracy of the docking predictions and to examine the critical interactions within the protein’s active site.^40^

#### 2.4.4 Molecular Dynamics

MD simulations were conducted to evaluate the interactions between marine pigments and their target proteins, with a particular focus on compounds exhibiting the lowest binding energy, indicative of a stronger binding affinity. These simulations were executed using the YASARA software suite (YASARA Bioscience GmBH, Vienna, Austria). The Amber14 force field was applied alongside periodic boundary conditions. The system’s temperature was set to 310 K, and the pH was maintained at 7.4. To neutralize the system, counterions (Na^+^ and Cl^–^) were added, along with TIP3P water molecules as the solvent. The simulations were run for 100 ns with a time step of 0.25 fs. Key parameters, including root-mean-square deviation (root mean square deviation (RMSD)), root-mean-square fluctuation (root mean square fluctuation (RMSF)), and radius of gyration, were monitored and analyzed at an interval of 25 ps.^42^

### 2.5 Measurement of Hemolymph Glucose Levels in *D. melanogaster*

A glucose level assay was performed to measure the hemolymph glucose concentration in each treatment group. For this assay, 70 third-instar *D. melanogaster* w^1118^ larvae from each control and treatment group were placed into separate Eppendorf tubes precleaned with 0.9% NaCl. The larvae were then homogenized using a microprobe, followed by centrifugation for 2 min. After centrifugation, 1000 μL of GOD-PAP reagent was added to 10 μL of the supernatant. The mixture was incubated for 10 min, after which the glucose concentration was measured using a spectrophotometer at a wavelength of 540 nm.^43^

### 2.6 Measurement of Length, Width, and Body Weight of *D. melanogaster* Larvae

The *w*^*1118*^ larvae were removed from the fly food and transferred to Petri dishes to facilitate the selection of third-instar larvae. For each of the five experimental groups, three third-instar larvae were individually weighed using an analytical balance (Sartorius), and the average body weight was then calculated.^44^ Additionally, the length and width of the larvae were measured by using an analytical caliper and microscope. The experimental groups included a control group with no treatment, a HSD group (30% sucrose), a HSD +31.25 μM CA group, a HSD +125 μM CA group, and a HSD +500 μM CA group.

### 2.7 Crawling Assay

Third-instar *w*^*1118*^ larvae were used for all treatments, with three larvae designated for each treatment group. Each larva was observed crawling on a glass Petri dish containing 20 mL of 2% agar medium, which was covered with paper boxes. The crawling activity of the larvae was quantified in millimeters per minute. During a 1 min observation period, the number of boxes traversed by each third-instar larva was recorded, with three replications for each treatment group.^45^

### 2.8 Survival Assay

Survival analysis was conducted to measure the time from larval emergence to maturation of adult flies. The *w*^*1118*^ larvae from both the control and treatment groups were placed in vials, with each vial containing 10 larvae. The larvae were then transferred to vials with either normal feed or treatment feed, maintained at 25 °C, with the feed changed every 3 days for each group. The experiment continued until all larvae had developed into adult flies. Observations were made to record the number of surviving larvae, pupae, and adult flies, continuing until all flies in the experimental groups had died.^46^

### 2.9 Gene Expression Analysis

Ten third-instar *D. melanogaster* larvae that had undergone treatment for 9 days were collected for RNA isolation using the Pure Link RNA Mini Kit following the manufacturer’s guidelines (Invitrogen, Thermo Fisher Scientific Inc., Massachusetts, USA). The expression levels of the target genes were analyzed by using the RT-qPCR method. The RT-qPCR assay was conducted in a 10 μL reaction volume utilizing SuperScript III Platinum SYBR Green One-Step RT-qPCR with ROX, following the manufacturer’s instructions (Invitrogen, Thermo Fisher Scientific Inc., Massachusetts, USA). The RT-qPCR runs were performed using specific primers for the target genes (Table 2) in a reaction volume of 10 μL, with the following cycling conditions: one cycle at 37 °C for 15 min, followed by 95 °C for 10 min, and then 40 cycles of 95 °C for 10 s, 60 °C for 30 s, and 72 °C for 30 s. A standard melt curve analysis was conducted for each RT-qPCR run to validate the specific amplification of the expected product. The host ribosomal protein gene, *rp49*, was used as the internal control, and its level was assessed using specific primers following a similar protocol applied to the target genes.

**Table 2:** Primers Used in the RT-qPCR Assay

genes	forward primer	reverse primer
*totA*	5′CCAAAATGAATTCTTCAACTGCT-3′	5′-GAATAGCCCATGCATAGAGGAC-3′
*srl*	5′-CTCTTGGAGTCCGAGATCCGCAA-3′	5′-GGGACCGCGAGCTGATGGTT-3′
*pepck*	5′-CCGCCGAGAACCTTATTGTG-3′	5′-AGAATCAACATGTGCTCGGC-3′
*rp49*	5′-CGCTTCAAGGGACAGTATCTG-3′	5′-AAACGCGGTTCTGCATGAG-3′

### 2.10 Data Processing and Statistical Analysis

Data from the hemolymph glucose levels, larvae body size, weight, and crawling assays were statistically analyzed using either Student’s *t*-test or One-Way ANOVA followed by post hoc analysis. The results are presented as mean ± standard deviation, with *p*-values less than 0.05 considered statistically significant. All data were processed and visualized using GraphPad Prism 9 (GraphPad Software, Boston, US).

## 3 Results and Discussion

### 3.1 CA Effectively Inhibits PTP1B Enzyme Activity in the In Silico Approach

**Figure 2:**
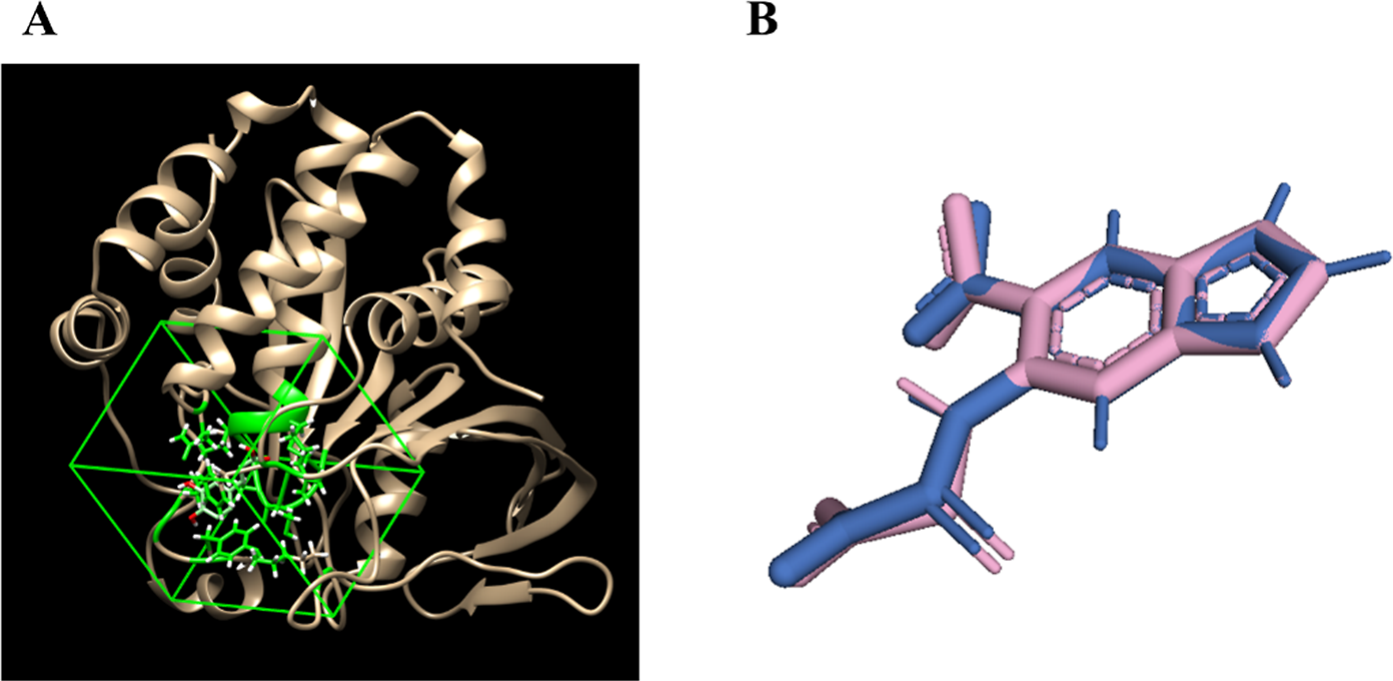
Grid box was positioned around the binding site of the PTP1B receptor (1C83) (A) and highlighting the redocked ligand (pink) and the native ligand (blue) (B), with an RMSD value of 0.001 Å.

In this study, the grid box parameters for the PTP1B receptor were set to dimensions of 16.9505 × 17.858 × 15.3153 with coordinates at 44.8165 × 14.2484 × 5.47854 (Figure 2A). Method verification was performed by calculating the RMSD of the ligand from the redocking process, yielding a value of <2 Å, as shown in Figure 2B, ensuring the reliability of the docking results.^47^

**Figure 3:**
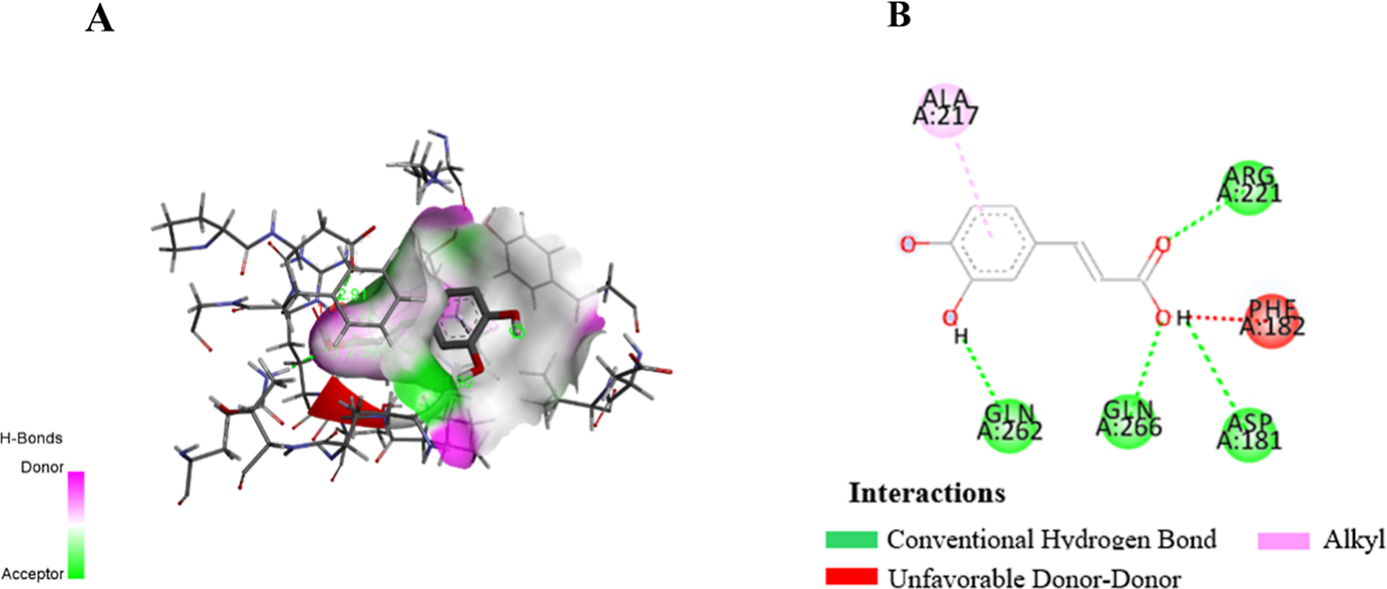
Docking view of CA in the binding site of PTP1B (PDB ID: 1C83). The stereo view of the docked complex between CA and 1C83 (A) and the interactions between CA and 1C83 presented in a 2D format (B). The diagram was created using Discovery Studio, with hydrogen bonds represented by green dashed lines along with their distances in Å, RMSD 0.159.

Molecular docking is a critical tool in drug discovery, as it predicts the interaction between a ligand and a target protein, offering insights into binding affinity, key residues, and mechanisms of action.^48^ In this study, in silico docking revealed that CA exhibited a stronger binding affinity to protein tyrosine phosphatase 1B (PTP1B) (Figure 3A), a key enzyme involved in glucose regulation, compared to the reference drug ertiprotafib. The results, as shown in Table 3, indicate that CA formed hydrogen bonds with key residues including ASP181, GLN262, and ARG221 (Figure 3B). Additionally, ASP181 and GLN262, which is also targeted by the native ligand,^49^ indicated a strong and specific interaction (Figure 3B). The amino acid residues ASP181, GLN262, and ARG221 play a crucial role in the inhibition of Protein Tyrosine Phosphatase 1B (PTP1B), a key regulator in insulin signaling and glucose metabolism. ASP181 is involved in the catalytic mechanism by forming hydrogen bonds that enhance the binding affinity of inhibitors,^50^ while GLN262 supports the structural stability of the active site, ensuring the optimal conformation for inhibitor interactions,^51^ ARG221 aids in substrate recognition through hydrogen bonding and electrostatic interactions, which are critical for inhibiting PTP1B activity.^50,51^ These findings suggest that CA more effectively stabilizes the PTP1B active site, thereby enhancing its inhibitory potential. This mechanism could improve insulin sensitivity and glucose uptake, positioning CA as a promising candidate for antidiabetic therapy.

**Table 3:** Binding Energy and Interaction Residues of CA against PTP1B (1C83)

	PTP1B (1C83)	
compound	Δ*G*	hydrogen bond
native ligand (PTP1B)	–8.5	**Arg221, Asp181, Gln262**, Ser216, Lys120, Gly220, Ala217, Ile219
caffeic acid	–7.0	**Arg221, Asp181, Gln262**, Gln266
ertiprotafib	–6.7	Tyr46

**Figure 4:**
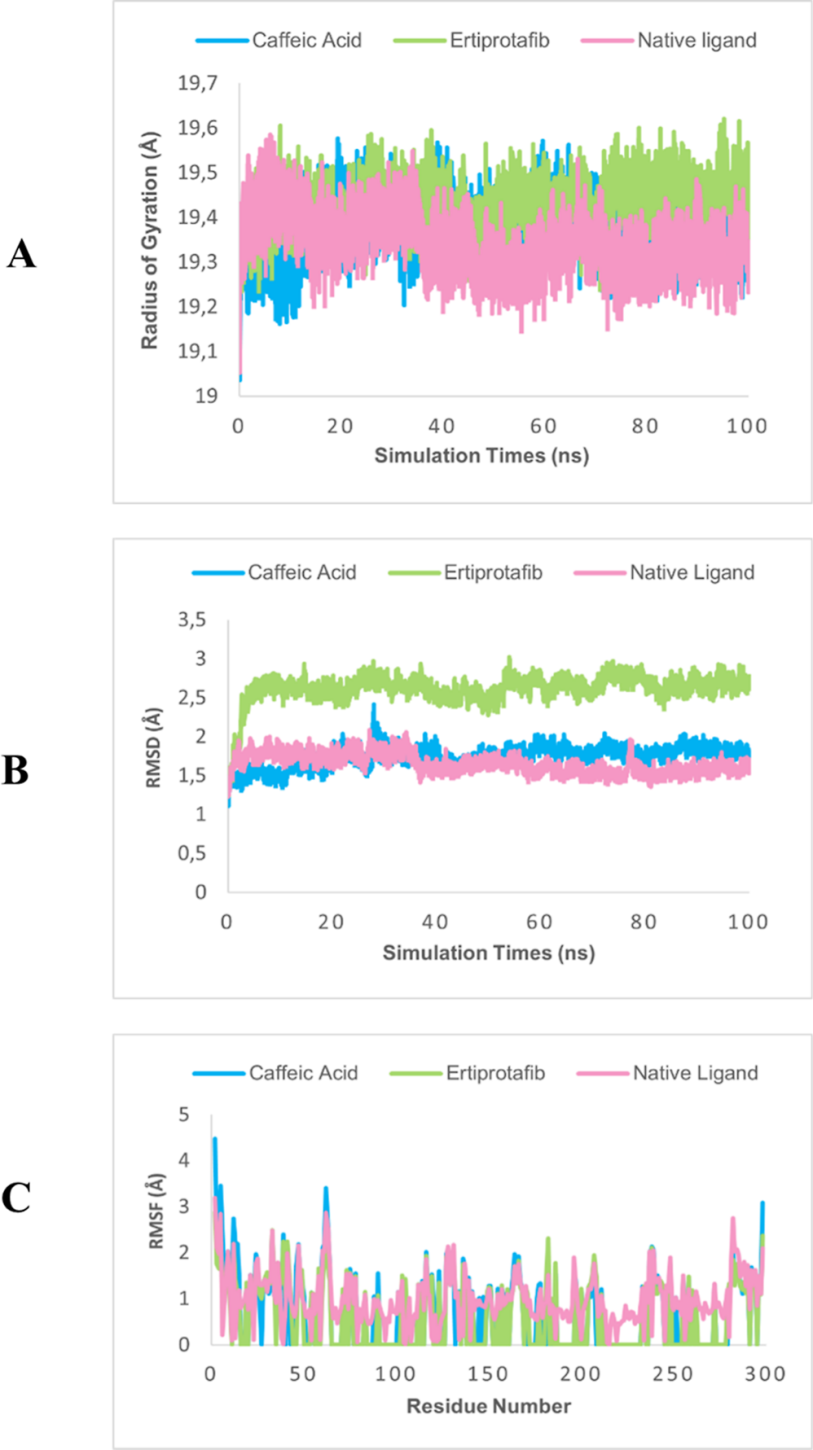
Molecular dynamics results radius of gyration (A), RMSD (B), and RMSF (C) of PTP1B.

The molecular dynamics results demonstrate that the PTP1B-caffeic acid complex exhibits superior structural stability compared to the positive control, ertiprotafib. Parameters such as the radius of gyration (Rg) indicate that the CA complex maintained a lower and more stable Rg value throughout the 100 ns simulation, suggesting a more compact and well-structured complex. In contrast, ertiprotafib displayed greater fluctuations, reflecting lower stability (Figure 4A). The RMSD analysis confirmed that the CA complex achieved stability more rapidly and maintained a more stable structure compared with ertiprotafib, while the natural ligand demonstrated stability comparable to that of CA. This indicates that CA not only interacts effectively with the active site of PTP1B but also forms an overall stable complex (Figure 4B). Additionally, RMSF analysis revealed that CA effectively stabilizes protein residues, exhibiting lower residue fluctuations compared to ertiprotafib, particularly in residues surrounding the active site. Meanwhile, the natural ligand also showed relatively low residue fluctuations, approaching the performance of CA in stabilizing protein residues (Figure 4C).

### 3.2 CA Reduces *D. melanogaster* Hemolymph Glucose Levels under Hyperglycemia Conditions

**Figure 5:**
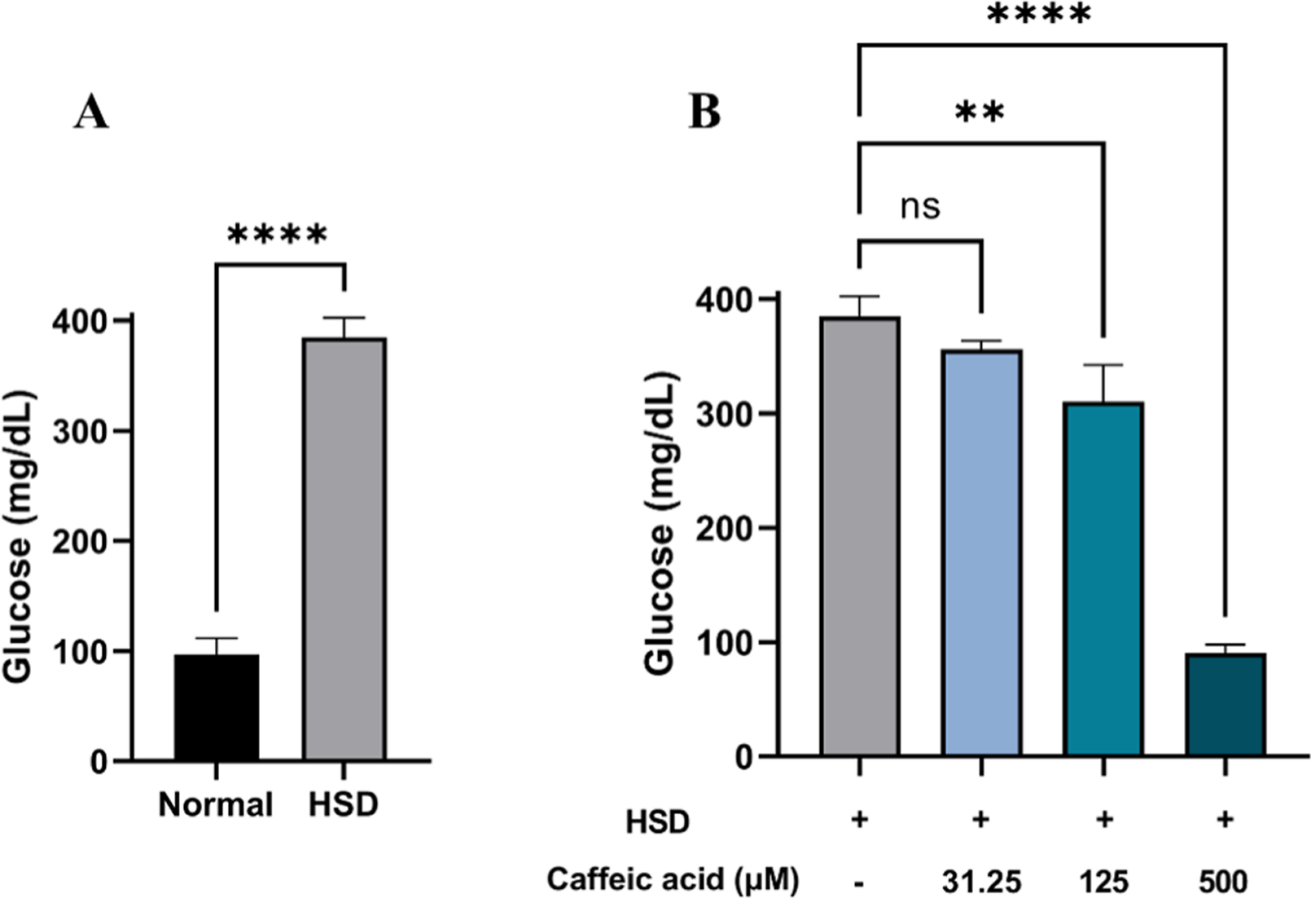
Measurement of hemolymph glucose level of *Drosophila melanogaster* after HSD treatment with or without CA. Comparison of larval hemolymph glucose levels between the normal control and HSD (A) and the comparison of glucose levels between HSD-fed larvae and those treated with varying concentrations of CA (B). Statistical significance is indicated by asterisks (**p < 0.01, ****p < 0.0001), with “ns” denoting no significant difference.

Hyperglycemia is characterized by elevated blood glucose levels and has been observed in both human and animal models such as the Zucker diabetic rat. Previous studies have demonstrated that high glucose concentrations lead to increased blood glucose levels,^52^ and similar outcomes were observed in *D. melanogaster*.^31,33^ To validate our in silico finding, we performed an in vivo experiment using the *D. melanogaster* model to assess the antihyperglycemic effect of CA. The antihyperglycemic properties of CA have been well documented in mammalian model organisms.^53^ In this study, we employed *D. melanogaster* to further explore its effects, not only assessing its presence or absence but also examining its long-term impact and potential complications, ultimately contributing to the development of a simplified model. If this model produces results consistent with those observed in rodent studies, then *D. melanogaster* could serve as a reliable alternative model. Previous research has successfully established a hyperglycemia model in *D. melanogaster*,^33,54^ which we tried to replicate in this study. The evaluation of hemolymph glucose levels in *D. melanogaster* larvae revealed a significant increase in glucose levels among those exposed to HSD (Figure 5A). In contrast, larvae treated with CA exhibited a substantial reduction in hemolymph glucose levels, with the most notable decreases observed at concentrations of 125 and 500 μM (Figure 5B). Using this model, we confirmed the antihyperglycemic properties of CA. These findings not only highlight *D. melanogaster* as a promising model for future investigations into diabetes-related complications and the long-term effects of antidiabetic treatments but also suggest that CA holds potential as a therapeutic agent for managing diabetes in humans.

### 3.3 CA Improves the Development of *D. melanogaster* under Hyperglycemic Conditions

**Figure 6:**
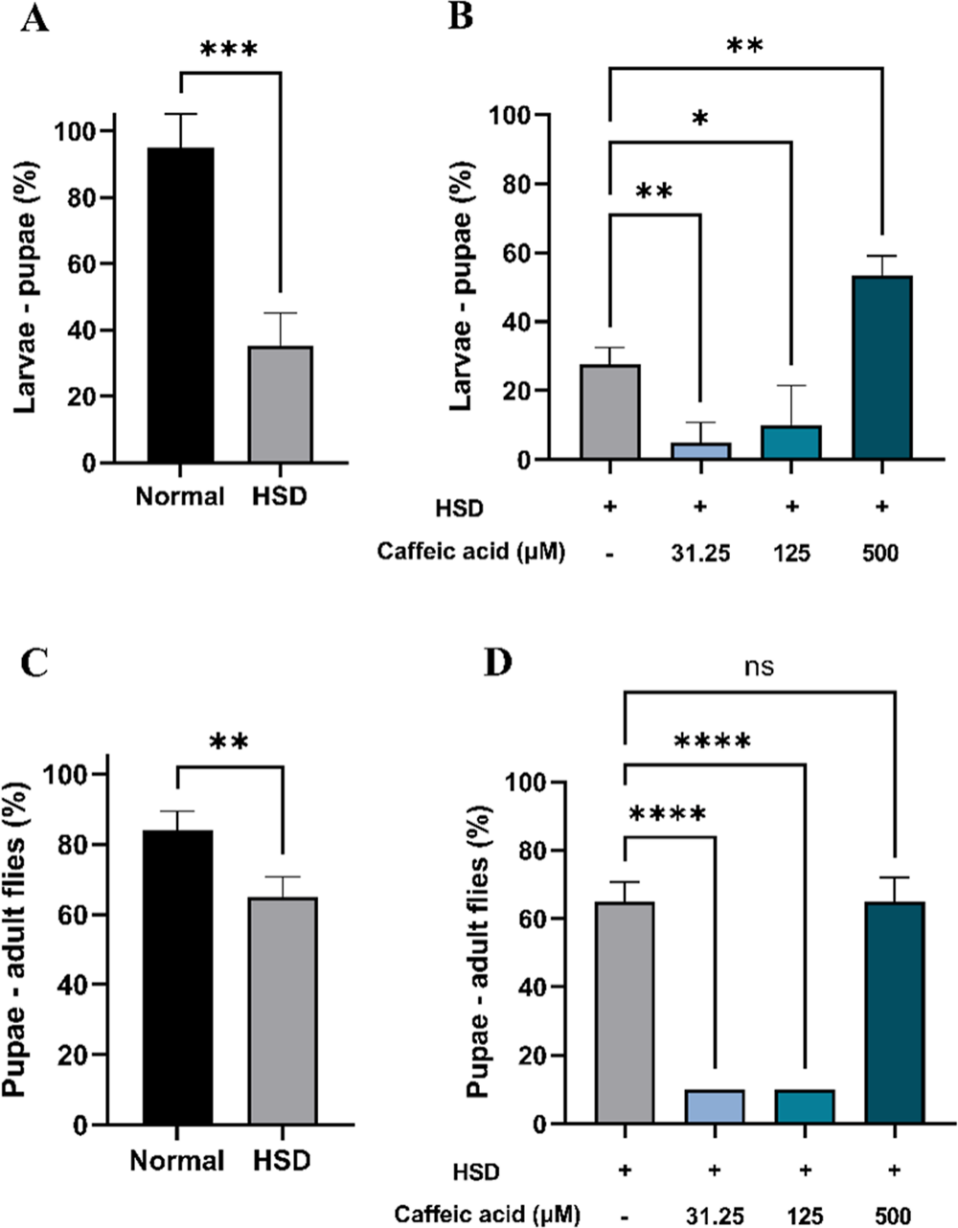
Improvement of *Drosophila melanogaster* survival at various developmental stages after HSD treatment in the presence or absence of CA. Comparison of larval-to-pupal survival between flies maintained on normal food and those on HSD (A), comparison of larval-to-pupal survival between flies maintained on HSD with and without CA treatment (B), comparison of pupal-to-adult fly survival between flies maintained on normal food and those on HSD (C), and comparison of pupal-to-adult fly survival between flies maintained on HSD with and without CA treatment (D). Statistical significance is indicated by asterisks (*p < 0.05, **p < 0.01, ***p < 0.001, ****p < 0.0001), with “ns” denoting no significant difference.

Hyperglycemia is a temporary condition characterized by excessively high blood sugar levels, typically caused by the frequent consumption of foods rich in carbohydrates or glucose and is consistently associated with reduced survival rates across different populations, including humans,^55^ mice,^56^ and *D. melanogaster*.^43^ As such, survival analysis can serve as an initial parameter to evaluate the potential antihyperglycemic activity of CA. The findings revealed that HSD at a 30% concentration had a detrimental impact on the growth and development of *D. melanogaster*, affecting both the larval-to-pupal transition (Figure 6A) and the pupal-to-adult stage (Figure 6C). These findings are in line with previous research by Loreto et al. (2021), which reported that a HSD can delay larval development. This delay arises from the inefficient utilization of glucose in the hemolymph by cells, resulting in an energy deficit that hinders growth and regeneration.^57^ However, supplementation with CA at three different concentrations significantly enhanced the development of HSD-fed larvae, as observed in their progression from larvae to pupae (Figure 6B) and from pupae to adult flies (Figure 6D). Notably, the highest concentration of CA (500 μM) resulted in the most substantial improvement.

### 3.4 CA Mitigates Body Size Reduction in *D. melanogaster* Larvae Induced by HSD Treatment

**Figure 7:**
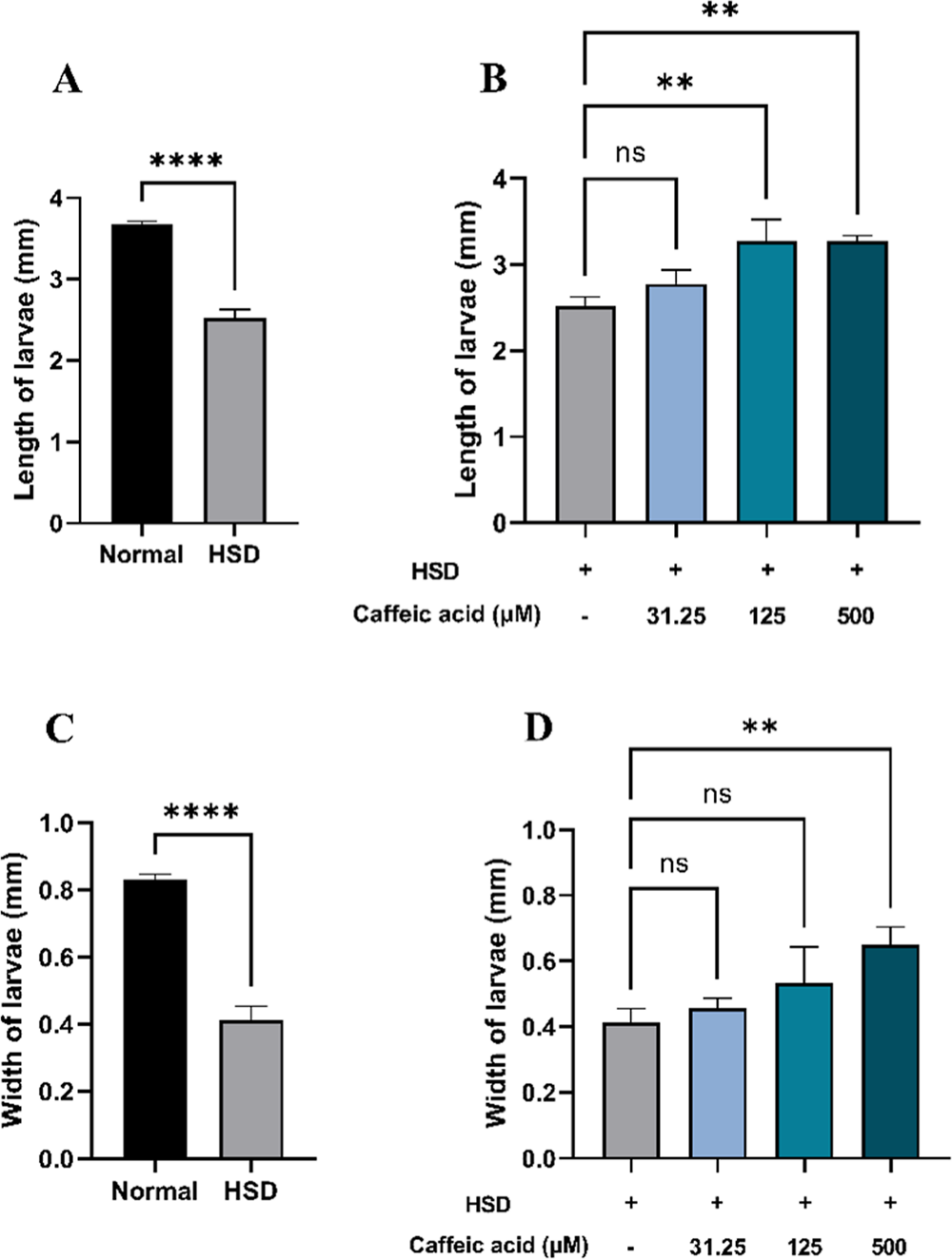
Measurement of larval length and width after HSD treatment in the presence or absence of CA. Comparison of larval length between normal control and HSD (A), comparison of larval length between HSD-fed larvae in the presence or absence of CA treatment (B), comparison of larval width between normal control and HSD (C), and comparison of larval width between HSD-fed larvae in the presence or absence of CA treatment (D). Statistical significance is indicated by asterisks (**p < 0.01, ****p < 0.0001), with “ns” denoting no significant difference.

Prolonged elevation of blood glucose levels due to hyperglycemia can lead to a reduction in the body size, as it disrupts normal metabolic processes and growth in *D. melanogaster*.^58^ In this experiment, a HSD significantly affected larval body size in those exposed to 30% sucrose, leading to a reduction in both length (Figure 7A) and width (Figure 7C). Consistent with previous studies, this reduction in the body size and growth inhibition might be attributed to impaired energy metabolism.^54^ However, HSD-fed larvae treated with CA at 125 and 500 μM exhibited significant improvements in both length (Figure 7B) and width (Figure 7D), approaching the values observed in the normal control. This suggests that CA may play a beneficial role in alleviating hyperglycemia-induced phenotypic abnormalities.

### 3.5 CA Improves Body Weight and Crawling Performance of *D. melanogaster*

**Figure 8:**
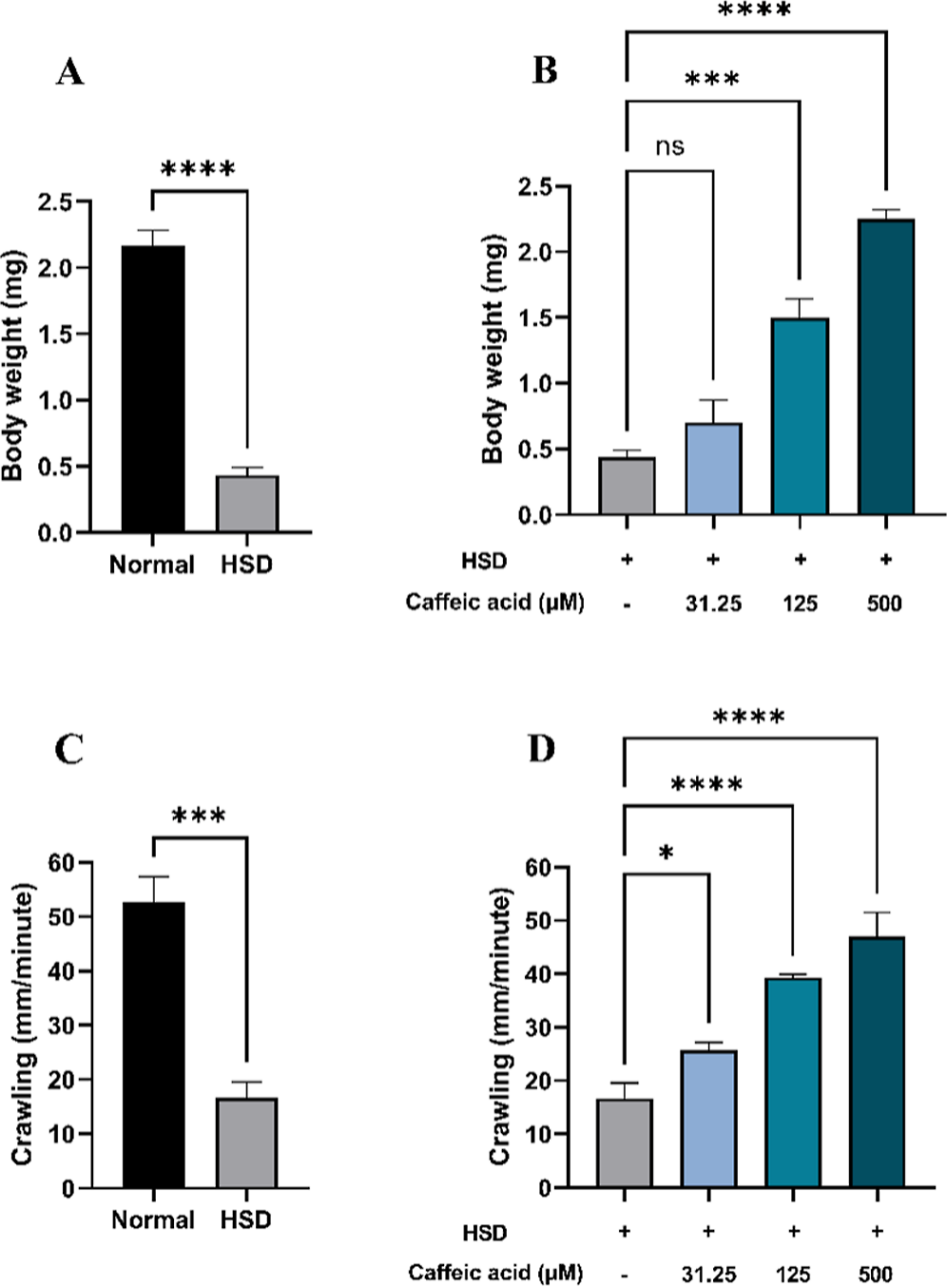
Measurement of body weight and larval crawling after HSD treatment in the presence or absence of CA. Comparison of larval body weight between normal control and HSD (A), comparison of larval body weight between HSD-fed larvae in the presence or absence of CA treatment (B), comparison of larval crawling between normal control and HSD (C), and comparison of larval crawling between HSD-fed larvae in the presence or absence of CA treatment (D). Statistical significance is indicated by asterisks (*p < 0.05, ***p < 0.001, ****p < 0.0001), with “ns” denoting no significant difference.

Hyperglycemia can lead to weight loss in humans and animals, including rats, as the body becomes unable to effectively utilize glucose, prompting the breakdown of fat and muscle for energy.^59^ Likewise, *Drosophila* larvae exposed to HSD exhibited a significant reduction in body weight (Figure 8A) and crawling/locomotor activity (Figure 8C). Under hyperglycemic conditions, the body fails to utilize glucose efficiently due to impairments in the insulin signaling pathway or resistance to insulin-like peptides, as observed in *D. melanogaster*. As a result, despite increased glucose levels in the hemolymph, cellular energy production is compromised, leading to an ATP deficiency that affects overall metabolism and growth.^33,57^ Conversely, treatment with CA at 125 and 500 μM significantly improved both body weight (Figure 8B) and crawling ability (Figure 8D), with the highest dose (500 μM) restoring these parameters to levels comparable to the normal control. These findings suggest that CA may contribute to the normalization of glucose utilization in *Drosophila* larvae.

### 3.6 CA Upregulates the Expression of Genes Regulating Metabolism and Nutrient Availability Response

**Figure 9:**
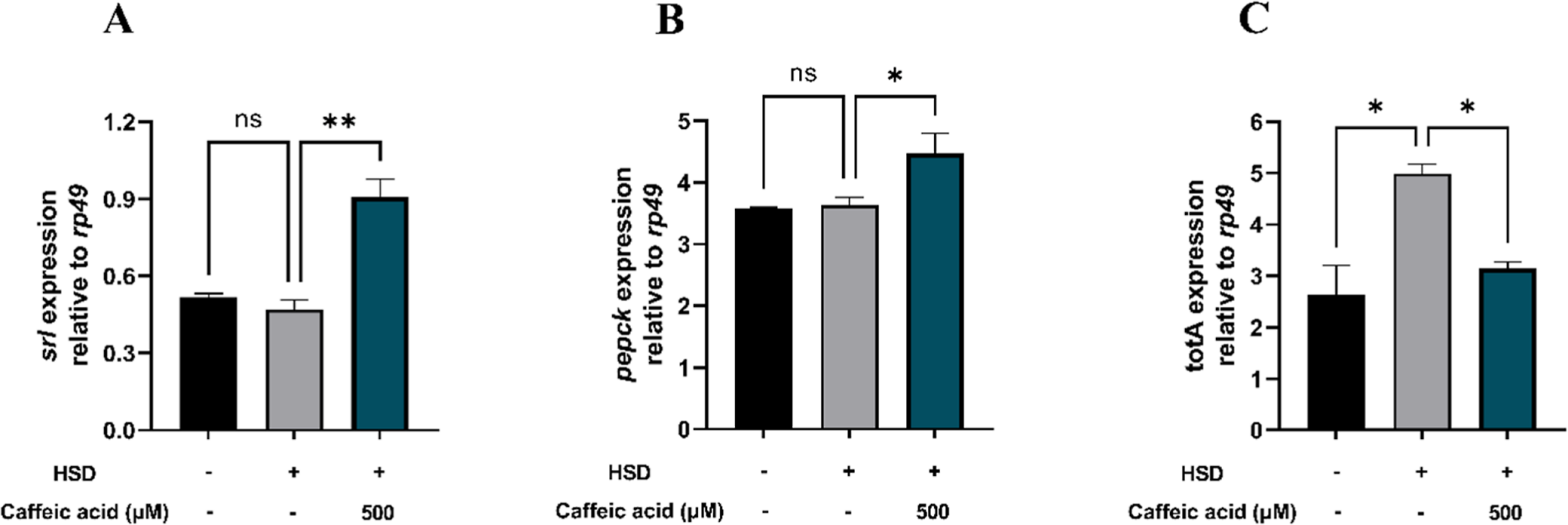
Measurement of *srl* (A), *pepck* (B), and *totA* (C) expressions following HSD treatment with or without CA. Treatment with CA at 500 μM significantly upregulated *srl* (A) and *pepck* (B) expression compared to the HSD control, while *totA* (C) expression was significantly reduced. Statistical significance is indicated by asterisks (*p < 0.05, **p < 0.01), with “ns” denoting no significant difference.

Hyperglycemia induces significant alterations in the expression of metabolism-related genes, particularly in the liver and retina of mice.^60^ Similarly, *D. melanogaster* subjected to a HSD exhibit an upregulation of genes involved in lipogenesis and gluconeogenesis, mirroring the mechanism of insulin resistance observed in humans.^61^ Additionally, hyperglycemia leads to the downregulation of genes associated with energy metabolism.^57^ The *srl* gene, which encodes the spargel protein in *Drosophila*, is homologous to PGC-1α in mammals. The spargel protein serves as a crucial regulator of mitochondrial biogenesis in both mammals and *Drosophila*.^62^ The increased expression of the *srl* gene observed in larvae treated with 500 μM CA suggests elevated levels of spargel, indicating enhanced mitochondrial performance in cellular respiration for energy production. This energy is vital for various activities in *Drosophila*, including movement. As illustrated in Figure 9A, larvae treated with CA at a concentration of 500 μM exhibited increased activity compared with those on a HSD. Moreover, the enhanced expression of the *srl* gene contributes to an increased lifespan in *Drosophila*, suggesting that the regulation of mitochondrial biogenesis through spargel significantly impacts the health and survival of organisms, including larval body size, as reflected in the measurements of length (Figure 7B) and width (Figure 7D). Thus, the spargel protein (encoded by *srl*) play essential roles in ensuring metabolic efficiency and the survival of *Drosophila* under hyperglycemic conditions, as depicted in Figure 9A.

The *pepck* gene encodes the enzyme phosphoenolpyruvate carboxykinase, which plays a crucial role in gluconeogenesis, the metabolic pathway responsible for synthesizing glucose from noncarbohydrate precursors, such as lactate, glycerol, and amino acids. Gluconeogenesis primarily occurs under glucose-deprived conditions, such as during fasting, and serves as a physiological response to counteract the deficiency of glucose required for energy production.^32^ The observed increase in the *pepck* gene expression following the administration of 500 μM CA may be linked to dietary restriction, which has been demonstrated to promote health and longevity across various species.^63^ In the context of dietary restriction, the body reacts as if it is experiencing glucose deprivation, leading to a reduction in the energy expenditure necessary for daily activities. This state necessitates the endogenous production of glucose via gluconeogenesis, a process stimulated by *pepck*. The synthesized glucose is subsequently utilized to generate adenosine triphosphate, the primary energy currency of cells,^32^ thereby influencing the larval size and body weight, as illustrated in Figures 7 and 8A. Thus, the *pepck* gene plays a vital role in maintaining glucose homeostasis, particularly under conditions of dietary restriction and low glucose availability, as depicted in Figure 9B.

The expression of the *totA* gene in *D. melanogaster* was significantly elevated in larvae fed HSD compared to the normal control (Figure 9C). This increase suggests that excessive sugar intake induces metabolic stress in *Drosophila*, triggering the activation of cellular defense mechanisms via the *totA* gene. Conversely, treatment with 500 μM CA resulted in a reduction in the *totA* gene expression when compared to the HSD control group (Figure 9C). This decrease implies that CA may exert protective effects, alleviating the metabolic stress caused by the HSD and potentially enhancing survival, as shown in Figure 6.

**Figure 10:**
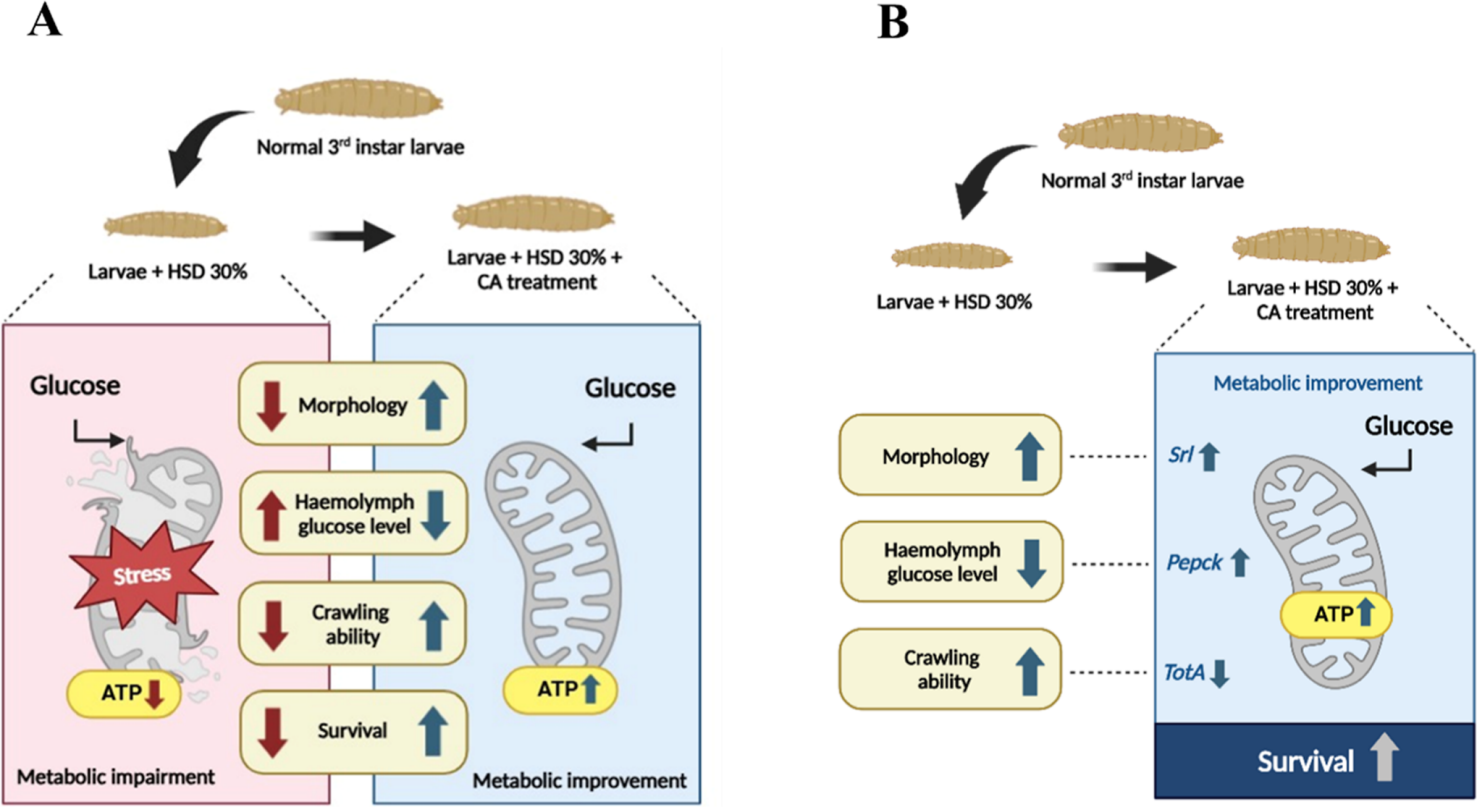
Illustration on the induction of hyperglycemia and CA treatment in *Drosophila melanogaster*. Larvae were exposed to a 30% sucrose solution to induce hyperglycemia, with phenotypic outcomes following CA treatment shown in (A). The correlation between phenotypic and molecular results after treatment is presented in (B). Created in BioRender. Nainu, F. (2025) https://BioRender.com/zbumoqj.

The molecular potential of CA in regulating glucose levels is associated with the expression of *srl*, *pepck*, and *totA* genes, which ultimately enhance mitochondrial ATP production and result in phenotypic improvements in the hyperglycemia model. Our hypothetic model can be seen in Figure 10.

## 4 Conclusions

In conclusion, CA demonstrates significant potential as an antihyperglycemic agent, exhibiting strong inhibitory activity against PTP1B in silico and effectively reducing glucose levels in a hyperglycemic *D. melanogaster* model. The observed improvements in physiological outcomes, including survival, body size, and movement, along with the regulation of genes involved in metabolism, suggest that CA may alleviate hyperglycemia through multiple mechanisms. These findings underscore its promise as a therapeutic strategy for managing hyperglycemia and its associated complications, warranting further investigation of its molecular mechanisms in advanced preclinical and clinical models.
